# Novel insights into ascorbate retention and degradation during the washing and post-harvest storage of spinach and other salad leaves

**DOI:** 10.1016/j.foodchem.2017.04.082

**Published:** 2017-10-15

**Authors:** Rebecca A. Dewhirst, Graham J.J. Clarkson, Steve D. Rothwell, Stephen C. Fry

**Affiliations:** a The Edinburgh Cell Wall Group, Institute of Molecular Plant Sciences, The University of Edinburgh, Daniel Rutherford Building, The King’s Buildings, Max Born Crescent, Edinburgh EH9 3BF, UK; b Vitacress, Lower Link Farm, St Mary Bourne, Andover, Hampshire SP11 6DB, UK; c wildFIRE Lab, Hatherly Laboratories, University of Exeter, Prince of Wales Road, Exeter EX4 4PS, UK^1^; d Edward Vinson Ltd, 4 Ewell Barn, Graveney Rd, Faversham ME13 8UP, UK^1^

**Keywords:** DCPIP, 2,6-dichlorophenolindophenol, DHA, l-dehydroascorbic acid, DKG, diketo-l-gulonate, HVPE, high-voltage paper electrophoresis, OxT, oxalyl-l-threonate, Vitamin C, Spinach, Salad leaves, Washing, Post-harvest storage, Chlorine, Oxalic acid

## Abstract

Post-harvest treatments of pre-packaged salad leaves potentially cause l-ascorbate loss, but the mechanisms of ascorbate degradation remain incompletely understood, especially *in planta*. We explored the extent and pathways of ascorbate loss in variously washed and stored salad leaves. Ascorbate was assayed by 2,6-dichlorophenolindophenol titration, and pathways were monitored by ^14^C-radiolabelling followed by high-voltage electrophoresis. All leaves tested showed ascorbate loss during storage: lettuce showed the greatest percentage loss, wild rocket the least. Spinach leaves were particularly prone to losing ascorbate during washing, especially with simultaneous mechanical agitation; however, washing in the presence of hypochlorite did not significantly increase ascorbate loss. In spinach, [^14^C]oxalate was the major product of [^14^C]ascorbate degradation, suggesting that commercial washing causes oxidative stress. This study highlights that ascorbate/dehydroascorbic acid are lost via the oxidative pathway during washing and post-harvest storage of salad leaves. Thus changes to washing procedures could potentially increase the post-harvest retention of ascorbate.

## Highlights

• Salad leaves lost 35–86% of their ascorbate during 10 d storage at 4 °C.
• Water-washing of spinach leaves and leaf discs increased ascorbate loss.
• Washing in presence of hypochlorite did not significantly increase ascorbate loss.
• Mechanical agitation of spinach leaves during washing exacerbated ascorbate loss.
• Oxalate was the major [^14^C]ascorbate by-product, indicating oxidative stress.

## 1 Introduction

Vitamin C, comprising l-ascorbate and dehydro-l-ascorbic acid (DHA), is chemically the simplest vitamin. Unlike humans, plants can synthesise ascorbate, accumulating it at up to millimolar concentrations such that it accounts for up to 10% of the total water-soluble ‘carbohydrates’ (Noctor & Foyer, 1998).

Vitamin C participates in collagen synthesis (Mandl, Szarka, & Bánhegyi, 2009). Other reported health benefits include the treatment or prevention of diabetes, cardiovascular disorders, age-related diseases and cancer (Ames et al., 1993, Mandl et al., 2009). Ascorbate is an antioxidant, but also has numerous other roles in plants including as an enzyme co-factor (Gallie, 2013), and in regulating the cell cycle (Smirnoff, Wheeler, & Loewus, 2000). In plants, apoplastic ascorbate may also play a beneficial pro-oxidant role, generating reactive oxygen species e.g. hydroxyl radicals (Fry, 1998), which may serve to loosen the cell wall during fruit ripening (Airianah, Vreeburg, & Fry, 2016).

Up to 90% of our dietary vitamin C is plant-derived (Lee & Kader, 2000) but cooking generally destroys much of the ascorbate in food (Lee & Kader, 2000). Therefore raw salads are an invaluable source of ascorbate. The ascorbate content of salad plants varies hugely, e.g. from 110 mg (curly kale) to as little as 3 mg (wholehead iceberg head lettuce) per 100 g fresh weight (McCance & Widdowson, 1991). Ascorbate content can also vary between cultivars of the same species (Hodges and Forney, 2003, Koh et al., 2012, Ren et al., 2013), and younger plant tissues often have higher ascorbate concentrations than older ones, e.g. in spinach (Bergquist, Gertsson, & Olsson, 2006) and celery (Huang et al., 2016), presumably related to the ascorbate’s role in plant growth.

The washing process of pre-packaged salads is also a potential source of ascorbate loss. Most commercial washing processes use recirculated water, treated with a sanitiser (e.g. chlorine-based). Iceberg lettuce washed in chlorinated water showed a marked decrease in ascorbate content after just one day’s storage compared with lettuce washed in non-chlorinated water (Kenny & O’Beirne, 2009). However, spinach leaves washed in chlorinated water did not show any rapid ascorbate loss (Karaca & Velioglu, 2014). Conversely, washing with chlorine-based sanitisers caused considerably more ascorbate loss during subsequent storage than washing in peroxyacetic acid-based sanitisers, although a water-only control was not included (Gómez-López, Marín, Medina-Martínez, Gil, & Allende, 2013). Equally, spinach washed in chlorinated water showed greater loss of antioxidant activity than when washed in oxalic acid (Cefola & Pace, 2015), probably owing to the oxidising nature of chlorine-based sanitisers. These somewhat contradictory results suggest a need for further investigation of chlorine effects in the spinach.

Other steps in the processing of pre-packaged salads could also lead to ascorbate loss. For example, the slicing sometimes used on iceberg lettuce influences ascorbate content throughout shelf life, with hand-torn leaves showing higher ascorbate retention than blade-cut leaves (Barry-Ryan & O’Beirne, 1999), presumably because blades cause more severe wounding, leading to ascorbate consumption during the wound response.

**Fig. 1:**
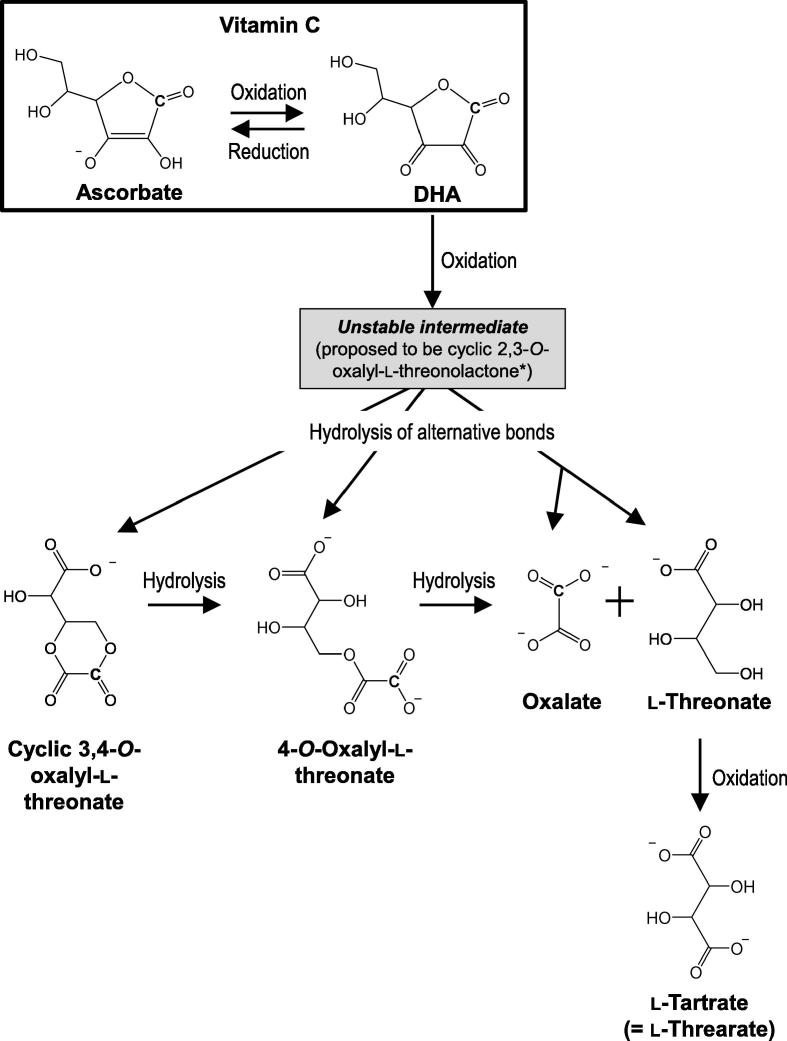
The oxidation pathway of ascorbate. Vitamin C consists of ascorbate (AA) and dehydroascorbic acid (DHA). Further degradation of DHA, e.g. by the oxidation reactions shown here, results in a loss of vitamin C activity. The initial oxidation step between AA and DHA is effectively reversible in plants owing to the presence of DHA reductase. The C shown in the structural formulae indicates the radiolabelled carbon derived from the [1-^14^C]AA used in this study. Pathway simplified from Parsons, Yasmin, and Fry (2011).

Although vitamin C has been widely studied for many decades, much remains unclear about its degradation pathways. The first relatively stable degradation product of ascorbate is DHA. The oxidation reactions involved are effectively reversible in plants owing to the presence of DHA reductase and monodehydroascorbate reductase (Foyer and Halliwell, 1977, Truffault et al., 2017). DHA can then be further oxidised to a range of products (Fig. 1), or hydrolysed to form diketogulonate (DKG), both these reactions representing a permanent loss of vitamin C from the plant tissue. DKG can itself be reduced to a redox-reactive substance with the formula C_6_H_6_O_5_ (Kärkönen, Dewhirst, Mackay, & Fry, 2017), and DHA can be further oxidised, e.g. to oxalyl threonate (OxT) and cyclic oxalyl threonate (Fig. 1) (Green and Fry, 2005, Parsons et al., 2011). Some plants accumulate ascorbate oxidation products, e.g. l-threarate (l-tartrate) in grapes (DeBolt, Hardie, Tyerman, & Ford, 2004) and oxalate in spinach (Yang & Loewus, 1975).

Increasing the ascorbate content of food (Hancock & Viola, 2005) would create more nutritious crops, as well as potentially making the crops themselves more tolerant of stress, such as oxidative stress. Although ascorbate can be easily synthesised chemically and then added to food, there is a general trend away from artificial food additives, creating a market for naturally ascorbate-enriched crops. An increase of ascorbate in crop plants could be achieved by either increasing the biosynthesis or decreasing the degradation. We are interested in the latter option. Therefore, this paper focuses on the post-harvest processing of young salad leaves as a potential area in which the loss of ascorbate could be minimised, as well as investigating the degradation pathways of ascorbate during post-harvest processing and storage.

## 2 Materials and methods

### 2.1 Plant material

Salad leaves used in experiments were grown commercially at Mullins Farm, Pewsey, Wiltshire and St Mary Bourne, Hampshire, UK, and processed on an industrial scale by Vitacress Salads Ltd, St Mary Bourne, from June to August 2013. The salad leaves studied were wild rocket (*Diplotaxis tenuifolia*), white wall ‘wasabi’ rocket (*Diplotaxis erucoides*), mizuna (*Brassica juncea var. japonica*), watercress (*Nasturtium officinale*), green Batavia (*Lactuca sativa*), iceberg lettuce (*Lactuca sativa*), spinach (*Spinacia oleracea*), red spinach (*Amaranthus dubius*), red chard (*Beta vulgaris,* subsp*. vulgaris*), pea shoots (*Pisum sativum*) and fennel (*Foeniculum vulgare*). In addition, a commercial variety (Toucan, from Rijk Zwaan) of spinach seeds was grown in soil at 21 °C (day) and 16 °C (night) with 16-h light levels of 150 µmol m^−2^ s^−1^. Leaves were harvested for experiments 4 weeks after sowing.

### 2.2 Determination of ascorbate content by titration with DCPIP

Salad leaves (1 g) were ground in 5 ml of either 2% (w/v) ‘metaphosphoric acid’ (an incompletely defined mixture of polymeric acids with overall empirical formula HPO_3_), 56 mM oxalic acid (Ponting, 1943) or 98 mM formic acid, with a pestle and mortar. The thoroughly ground sample was then vacuum-filtered on Whatman No. 1 filter paper and the filtrate collected, or the samples were centrifuged at 1000*g* for 10 min and the supernatant collected. Duplicate 1-ml aliquots of the filtrate or supernatant were titrated with 3.73 mM DCPIP (2,6-dichlorophenolindophenol), added in 10-µl shots until a pink colour remained for 10 s. The volume of DCPIP added was compared to a standard curve of ascorbic acid concentrations.

### 2.3 Postharvest washing procedures

Salad leaves harvested from Vitacress farms were stored in unsealed plastic packaging in the dark at 4 °C for up to 10 days. Watercress (*Nasturtium officinale*), spinach (*Spinacia oleracea*) and wild rocket (*Diplotaxis tenuifolia*) from the same harvest batch were sampled before and after the commercial washing process. The washing process at Vitacress consists of a counter-current flow system in which the leaves are washed in spring water. The leaves are spun dry and distributed into plastic packaging (microperforated 30-µm orientated polypropylene).

The industrial washing process was also simulated under laboratory conditions. Samples of spinach leaves (1 g, in triplicate) were incubated either in air (with no H_2_O added), in still water or in shaken water (on a small orbital shaker) for 0.5, 1 or 2 h in the dark, then analysed for ascorbate. Samples (1 g in triplicate) were also analysed immediately after harvesting (time 0).

The effect of chlorine on the retention of ascorbate in spinach leaves during washing was investigated. Samples of spinach leaves purchased from a local supermarket (1 g in triplicate) were incubated in plastic vials either in air (with no H_2_O added), in H_2_O or in chlorinated H_2_O (100 ppm active chlorine from sodium hypochlorite), all with gentle shaking at 7 °C for 1 h in the dark.

### 2.4 Infiltration of spinach leaves with [^14^C]ascorbate

Each of three spinach leaves was placed with its petiole (cut at 90° with a razor blade) in a round-bottomed tube containing l-[1-^14^C]ascorbate (8 kBq; Amersham Pharmacia Biotech UK Ltd) diluted to 50 µl with H_2_O. The [^14^C]ascorbate was taken up into the leaf by transpiration. After this initial solution has been taken up, three further aliquots (50 µl) of H_2_O were added into the tube, to ensure all the [^14^C]ascorbate was taken up. The presence of radioactivity in the lamina was confirmed with a Geiger counter. Leaf discs (1 cm diameter) were cut out from the lamina, avoiding the main veins (as shown in Fig.6a). Sets of four equivalent discs were prepared and each disc within a set was subjected to a different treatment: analysed immediately (time 0) or incubated in either air, 5 ml still water or 5 ml shaken water (on a mini orbital shaker at 150 rpm with an orbit of 2 mm) at 7 °C. The two halves of the lamina are assumed to be identical, thus each disc within a set was presumed to contain equivalent levels of radiolabelled compounds. This allowed direct comparisons to be made between these four leaf discs.

**Fig. 2:**
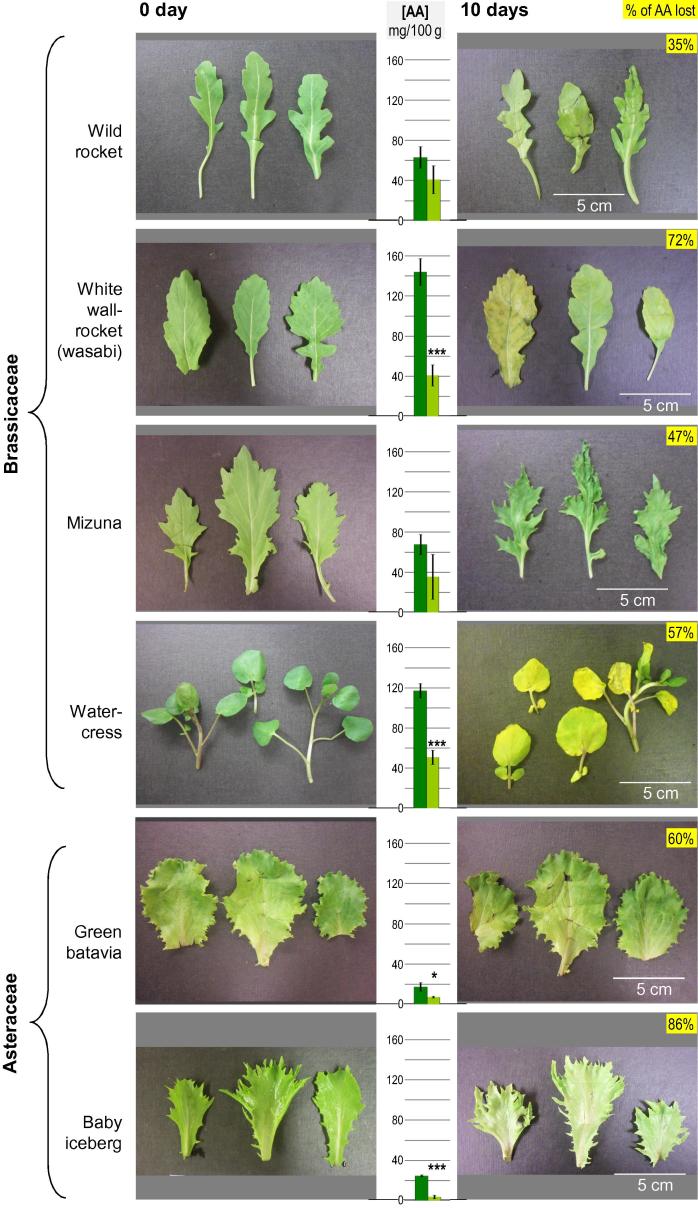

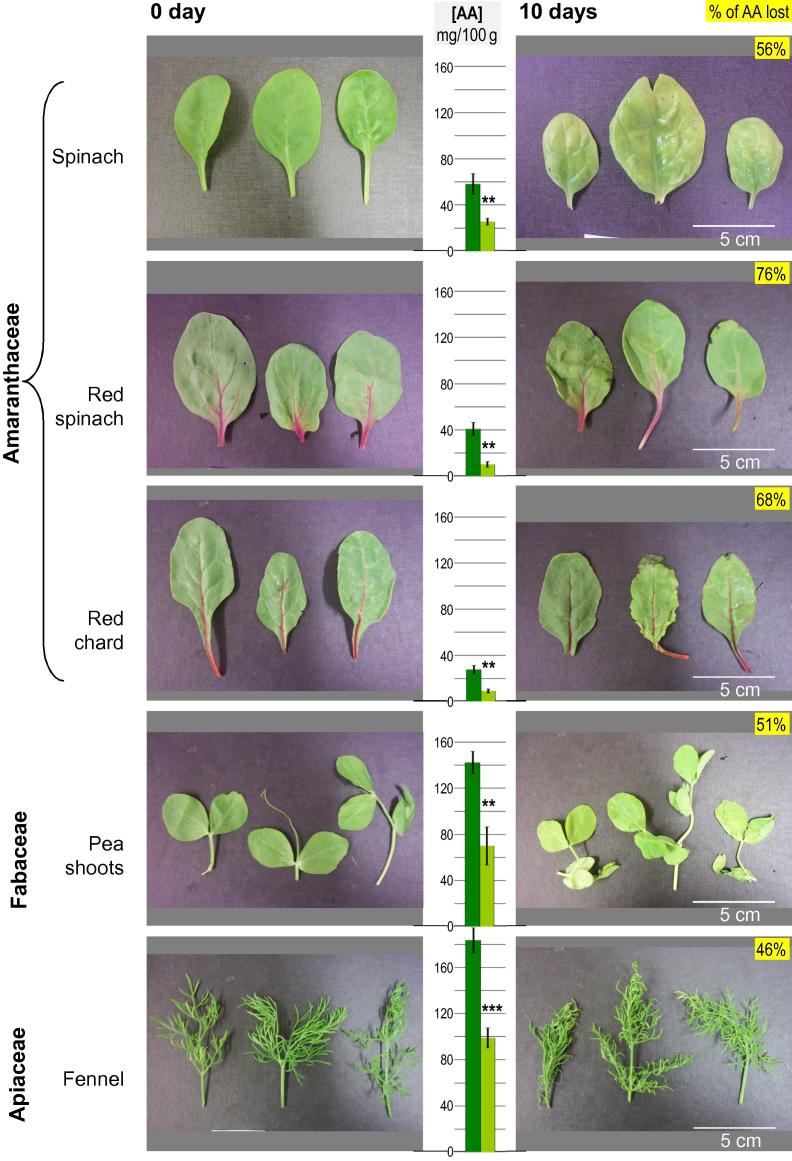
Changes in visual characteristics and ascorbate loss in different salad leaves during storage. Various types of salad leaf were stored in the dark at 4 °C for 10 days, then photographed and assayed for ascorbate content. The histograms show mg ascorbic acid per 100 g fresh weight after 0 and 10 d (left and right bar, respectively). Each bar shows the mean of titrations of 6 separate extracts ± SE. Significant differences between 0 and 10 d are indicated (Student’s *t*-test; ^*^p < 0.05, ^**^p < 0.01, ^***^p < 0.001, n = 6). Values in yellow boxes indicate the % loss of ascorbate between 0 and 10 d.

**Fig. 3:**
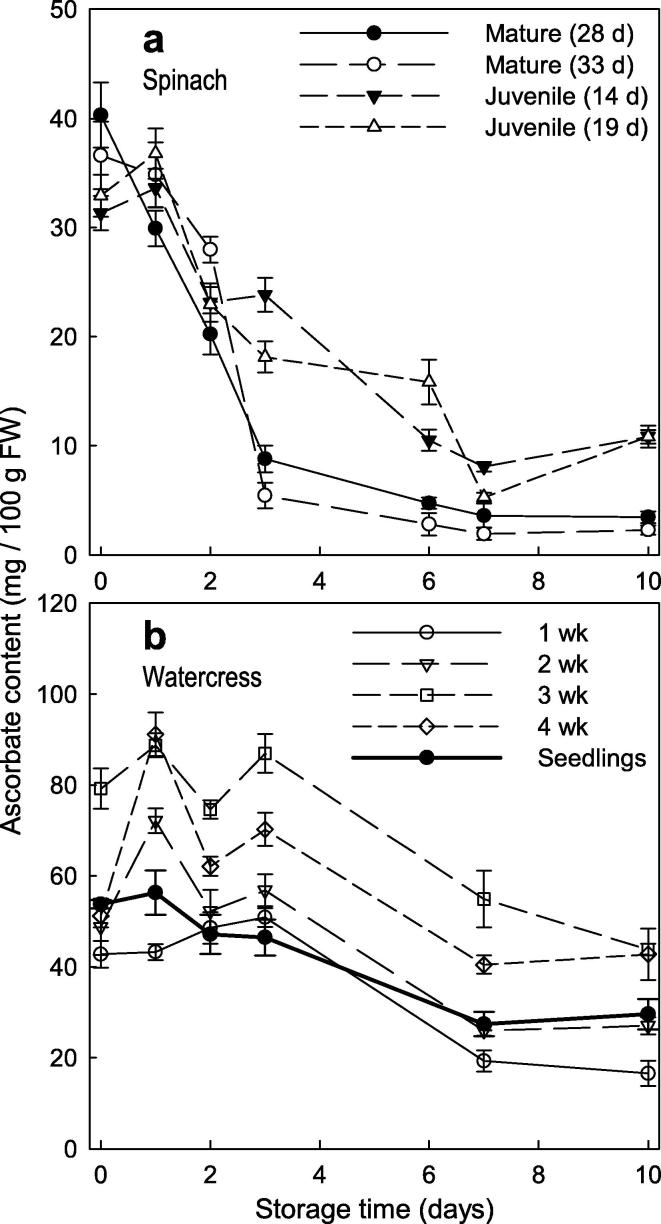
Ascorbate loss in different growth stages of spinach and watercress. Plant material tested was: (a) Four batches of spinach (cultivar Toucan) were simultaneously harvested, at different growth stages: two relatively late (harvested 28 and 33 d after field sowing, at the ‘vegetable-leaf’ stage; described here as ‘mature’ spinach) and two relatively early (harvested 14 and 19 d after sowing, at the ‘baby’ salad-leaf stage; described here as ‘juvenile’ spinach). (b) Watercress seedlings (7–10 days after sowing), and plants harvested 1–4 weeks after these seedlings had been planted out. In each case, detached leaved were stored in the dark at 4 °C and assayed for ascorbate content after 0–10 d. Each data-point represents the mean of three leaf samples ± SE.

**Fig. 4:**
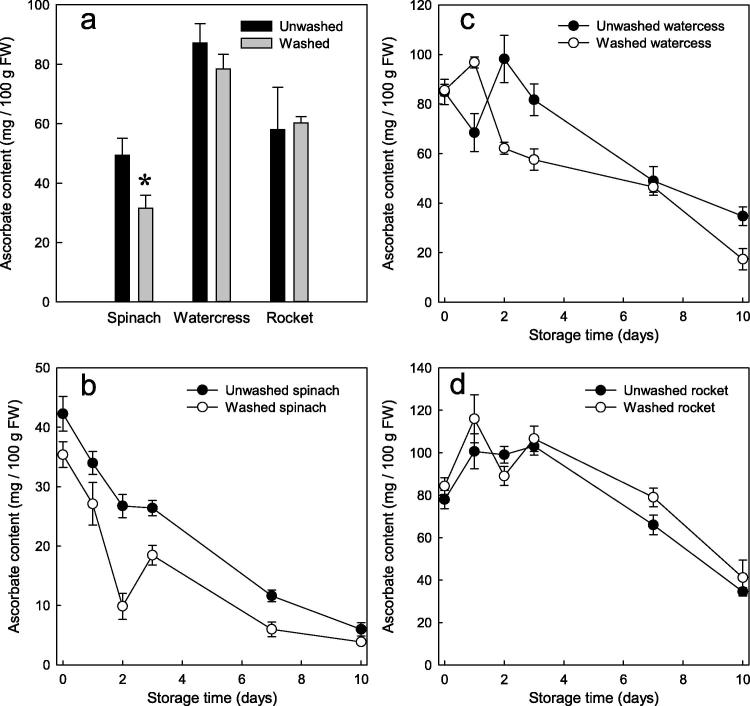
Ascorbate content of salad leaves before and after washing and/or subsequent storage. (a) Salad leaves (spinach, watercress and wild rocket) from the same harvest batch were assayed for ascorbate before and immediately after the washing process. Data show the mean ± SE (n = 6); ^*^ = significant effect of washing (Student’s *t*-test; p < 0.05, n = 6). (b)–(d) Samples of washed and unwashed spinach (b), watercress (c) and rocket (d) leaves were then stored in the dark at 4 °C in unsealed plastic packaging (standard commercial barrier salad film consisting of microperforated 30-μm orientated polypropylene, 80 g fresh weight per 25 × 20-cm bag) for up to 10 days. The ascorbate content was assayed at intervals. Data are mean ± SE (n = 3).

**Fig. 5:**
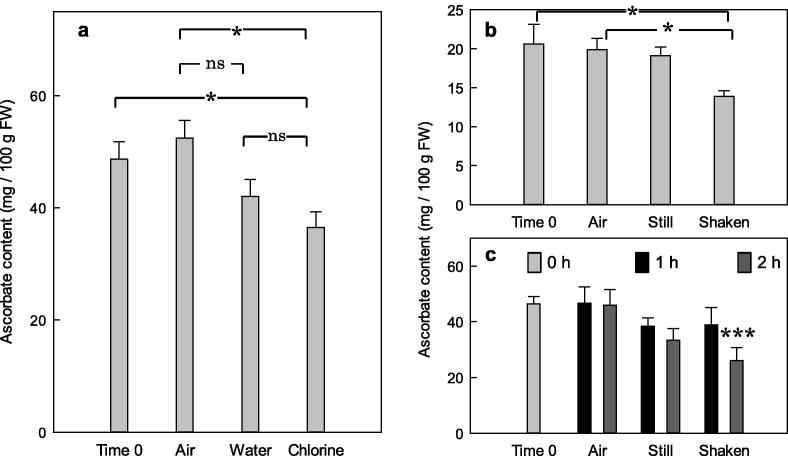
Effect of chlorine and mechanical agitation on ascorbate loss during washing of spinach. (a) Effect of chlorine. Spinach leaves (1 g per sample, from a local supermarket) were incubated for 1 h either in air, or submerged in 20 ml H_2_O (water), or submerged in 20 ml chlorinated H_2_O (100 ppm active chlorine). The samples incubated in pure and chlorinated water were incubated shaken on a mini orbital shaker (150 rpm, with a 2-mm orbit) for 1 h. Ascorbate was determined initially (time 0) and after the 1-h incubation. Data show the mean (of 12 replicate leaf samples) ± SE. Statistically significant differences between conditions are indicated (one-way ANOVA, *n* = 12): ^*^p < 0.05; ns, difference not significant (*p* > 0.05). (b, c) Effect of mechanical agitation on leaves and leaf-discs. Whole spinach leaves (1*g* fresh weight) (b) or 1-cm-diameter leaf discs (250 mg fresh weight) (c) were incubated in 60-ml plastic beakers at 7 °C in the dark, either in air or submerged in 5 ml H_2_O (still water) or submerged in 5 ml H_2_O and shaken on a mini orbital shaker (150 rpm with an orbit of 2 mm) (shaken water) for 1 or 2 h, and then assayed for ascorbate. Data show the mean from three separate beakers ± SE. Significant differences from the corresponding time-0 sample are indicated (one-way ANOVA, ^*^p < 0.05, ^***^p < 0.001, n = 3).

**Fig. 6:**
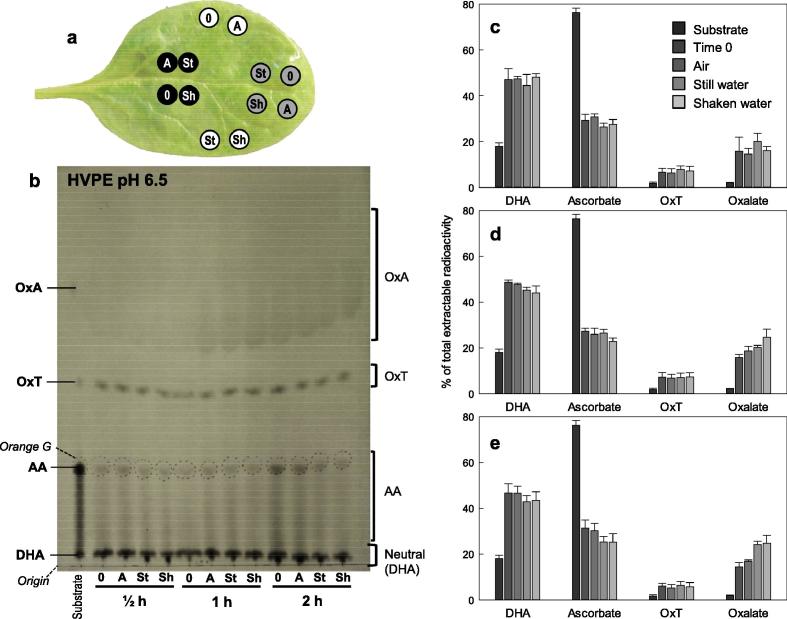
The degradation of [^14^C]AA in spinach leaf discs during washing. Three individual excised spinach leaves were fed [^14^C]AA (50 µl; approximately 0.5 mM) through the cut petiole. Twelve discs were then punched from each lamina as shown in (a) (black, proximal; grey, distal; white, lateral), and treated for 0.5, 1 or 2 h at 7 °C in the dark in 20-ml plastic vials containing only air (A), 5 ml of still water (St), or 5 ml of shaken water (Sh). Time 0 is represented with ‘0’. Extracts from each of the 36 leaf discs were then run by HVPE at pH 6.5 (12 samples and a substrate-only control per sheet), and the papers was autoradiographed; a representative set of 12 is shown in (b). The radioactivity present in each spot was also quantified by scintillation counting (c–e, for 0.5, 1 and 2 h incubation respectively). Quantification of ascorbate includes the streak (visible in b), which comprises (electrophoretically mobile) AA that oxidised to (immobile) DHA during the electrophoresis. The bars in c–e each represent the mean of three individual leaf disc extracts ± SE; to avoid bias, the three replicate discs used for each average came from all three leaves and represented all three zones — for example, leaf-1 distal, leaf-2 proximal and leaf-3 lateral.

Five replicate sets (of the four treatments) of leaf discs were prepared for each of the three time-points (0.5, 1 and 2 h). After the appropriate incubation time the leaf disc was removed from the vial and the radiolabelled compounds were extracted in 0.5% formic acid (200 µl) with a mortar and pestle. The extract was stored at −80 °C until further analysis.

### 2.5 Analysis of radiolabelled extracts

The extracts were centrifuged at 2000*g* for 5 min, and samples (50 µl) of the supernatant were run by HVPE (high-voltage paper electrophoresis) in pH 6.5 buffer (pyridine/ acetic acid/ H_2_O, 33:1:300 v/v/v) for 30 min at 2.5 kV or in pH 2.0 buffer (formic acid/acetic acid/ H_2_O, 1:4:45, v/v/v) for 50 min at 2.5 kV.

The presence of [^14^C]ascorbate and any degradation products formed was detected by autoradiography (3 week exposure to Kodak BioMax MR-1 film) or scintillation counting. Samples dried onto paper were assayed in 2 ml ScintiSafe scintillation fluid in a Beckman LS 6500 CE multi-purpose scintillation counter.

### 2.6 Statistical analyses

Statistical significance (p > 0.05) of the ascorbate content of salad leaves was assessed using Student’s *t*-test or one-way analysis of variance (ANOVA) with a post-hoc Tukey test to determine the differences between treatments.

## 3 Results

### 3.1 Ascorbate retention during salad shelf-life

The aim of this work was to determine changes in the concentration of ascorbate during the washing and postharvest storage of a range of packaged salad leaves. The mean initial ascorbate concentration among the eleven salad types surveyed was 80 mg/100 g fresh weight (∼4.7 mM in total sap), but there was an 11-fold difference in initial ascorbate concentration between the highest (fennel) and lowest (Green Batavia lettuce) (Fig. 2, histograms). Regardless of its initial concentration, endogenous ascorbate proved unstable during 10 days’ storage at 4 °C in the dark. All types of salad leaf tested showed a significant decrease in ascorbate over the 10 days, apart from wild rocket and mizuna; however, even these showed a trend towards ascorbate loss, albeit not statistically significant (Fig. 2). The mean ascorbate loss during the 10-d period was 59%, the extreme being an 86% loss in baby iceberg lettuce. There was no significant correlation between initial ascorbate content and the proportion lost during leaf storage (r^2^ = 0.142, n = 11; p > 0.1; Fig. S1).

The decline in visual quality during storage varied greatly between the different types of salad leaf. For example, fennel and pea shoots showed very little change in colour and negligible bruising (Fig. 2), and these two species were among the highest in ascorbate content. However, two other high-ascorbate species, watercress and ‘wasabi’ (white wall rocket; *Diplotaxis erucoides*), did show dramatic changes in colour, with obvious yellowing occurring during storage, so there was no consistent correlation between ascorbate content and visible deterioration. Equally, the other members of the Brassicaceae showed clear signs of bruising during storage, and the Amaranthaceae species show discolouration and slight bruising (Fig. 2).

### 3.2 Pre-harvest factors affecting ascorbate retention

Pre-harvest factors, such as growth stage, have been reported to affect the ascorbate content of salad leaves (Bergquist et al., 2006). Therefore, of the eleven salad types surveyed in Fig. 2, we selected two species to investigate this aspect. In addition, we determined the kinetics of ascorbate loss during storage rather than only the 10-d end result. The ascorbate content of excised spinach leaves (two batches harvested at juvenile growth stage and two at mature growth stage) and watercress leaves (from seedlings and from developing plants harvested 1–4 weeks after the seedlings had been planted out) was monitored throughout 10 days’ storage.

The initial ascorbate concentration of the spinach samples was 35 ± 4 mg/100 g FW (mean ± SD of the four batches representing two growth stages; n = 4), corresponding to ∼2.0 mM in total sap (Fig.3a). The loss of ascorbate occurred more quickly and more extensively in mature- than in juvenile-stage spinach. After 3 d of storage, mature spinach had lost about 80% of its ascorbate, whereas juvenile spinach had lost only 25–45%. Even after 10 d, juvenile spinach leaves still contained 30% of the initial ascorbate, whereas the mature spinach had only 10% remaining (Fig.3a). The visual quality of juvenile spinach leaves after 10 d of storage was also better than in mature spinach, which showed more bruising and discolouration (Fig. S2a, b). Thus, ascorbate loss (but not initial ascorbate concentration) correlated with visual deterioration.

Commercially, watercress is grown from seedlings planted in water-beds, and is generally harvested three weeks after planting out. The initial ascorbate concentration of the watercress samples was 1.2–2.2× higher than in spinach (Fig.3b). Watercress plants harvested 3 and 4 weeks after planting out had a higher ascorbate concentration than the younger samples. At all ages the ascorbate content remained fairly stable for the first 3 days of storage.

Thereafter, all samples gradually lost ascorbate at about the same absolute rate (∼7 mg/100 g FW per day), so the younger samples lost a higher percentage of their initial ascorbate per day. The data suggest that the current practice of harvesting watercress at three weeks after planting is the optimum in terms of ascorbate content.

In watercress, the final ascorbate content after 10 d storage was 58 (±16) percent (mean ± SD) of the initial content, whereas in spinach it was ∼34% and only ∼7% for juvenile and mature plants respectively (Fig. 3). Thus watercress contained more ascorbate, and lost less, than spinach. Despite this, the watercress suffered visual deterioration, especially in terms of chlorophyll loss (Fig. S2b). The mature-stage spinach showed serious bruising after 10 days’ storage, whereas the juvenile-stage spinach, with its greater ascorbate retention, resisted bruising (Fig. S2a).

### 3.3 Post-harvest processes affecting ascorbate retention

The salad washing process at Vitacress involves a counter-current flow system with leaves loaded at the water-outflow end of a wash-tank and exiting at the fresh spring-water inlet end. The leaves are submerged in the water, and undergo air-generated turbulence to remove particles of soil and grit. The leaves are then spun surface-dry, before being packaged in polypropylene and distributed to supermarkets. We therefore tested whether this washing process could potentially be causing a loss of ascorbate.

Spinach, watercress and wild rocket were assayed for ascorbate before and after the washing process (Fig.4a). Spinach leaves showed a significant decrease (Student’s *t*-test, p < 0.05, n = 6) in ascorbate after commercial washing, whereas ascorbate in watercress and wild rocket showed no significant change (Fig.4a).

The washed and unwashed samples were stored for up to 10 days in plastic packaging in the dark, and assayed for ascorbate at intervals. The unwashed spinach showed consistently higher ascorbate levels than the washed spinach (Fig.4b). Interestingly, as seen in Fig. S2, ascorbate in spinach began to decrease (at ∼4 mg/100 g FW per day) immediately upon storage, whereas in watercress (Fig.4c) it remained fairly stable for 3 days, before beginning to decline. Wild rocket (Fig.4d) resembled watercress in this respect.

It is common practice in the pre-packaged salad industry to wash leaves in chlorinated water (typically 20–60 ppm active chlorine). However, at Vitacress Salads Ltd, the leaves are washed in single pass spring-water only. As chlorine is an oxidising agent, it is possible that washing leaves in the presence of chlorine would lead to a loss of ascorbate. Various sanitizers have been compared (Gómez-López et al., 2013), but pure water has rarely been compared with these. The effect of washing in chlorinated water on the ascorbate content of spinach compared with washing in pure water was therefore investigated (Fig.5a). Spinach leaves shaken in chlorinated water showed a significant (p < 0.05, one-way ANOVA) decrease in ascorbate content compared with those incubated, stationary, for an equivalent time in air (Fig.5a). Leaves shaken in chlorinated water appeared to suffer a larger and more significant ascorbate loss than those in pure water; however, the difference between chlorinated and pure water was not significant.

We further investigated the loss of ascorbate in spinach leaves to determine where in the washing process the ascorbate was predominantly lost. Spinach leaves submerged in turbulent water showed a significant (p < 0.05, one-way ANOVA) loss of ascorbate, compared with leaves at time 0 and those incubated in air (Fig.5b). That there was no significant difference between time 0 and leaves incubated in still water suggests that mechanical stress causes a loss of ascorbate. This difference was replicated with spinach leaf discs, although the ascorbate was more readily lost in discs than in whole leaves (Fig.5c). This may be due to ascorbate being consumed during the wound response in the excised discs, the preparation of which would be likely to elicit some wounding.

### 3.4 Ascorbate degradation pathways

A loss of ascorbate during washing has been previously reported in spinach (Gómez-López et al., 2013), but the fate of this lost ascorbate had not been investigated. The degradation pathways of ascorbate have not been fully elucidated, particularly not *in planta*. The first relatively stable oxidation product of ascorbate, DHA, can undergo either further oxidation followed by various hydrolytic steps (Fig. 1) or direct hydrolysis (to diketogulonate and its downstream products, not shown in Fig. 1; Green and Fry, 2005, Kärkönen et al., 2017, Parsons et al., 2011) — in either case producing irreversible degradation products. Identifying the products formed from DHA in washed leaves would enable us to determine which degradation pathway(s) the vitamin C had taken, and thus potentially provide information about the nature of the stress causing vitamin loss during washing.

We monitored the fate of ascorbate during the washing process in spinach leaves by using radiolabelled ascorbate. The nature of the experiment required the use of leaf discs rather than whole leaves. Discs were taken from a spinach leaf that had been incubated with [^14^C]ascorbate. HVPE analysis of extracts of the discs after various severities of washing revealed oxalate to be the major ascorbate degradation product (Fig. 6). Spinach is well known to accumulate relatively high levels of oxalate (Taylor and Curhan, 2007, Yang and Loewus, 1975) and our finding confirms the role of ascorbate as a precursor to oxalate in spinach leaves. The yield of [^14^C]oxalate increased over time, correlating with a loss of ascorbate and DHA. The samples incubated for 2 h showed the greatest increase in [^14^C]oxalate (Fig.6d). This increase in [^14^C]oxalate was greater in the shaken samples than in the still samples at 1 h, but fairly similar at both 0.5 h and 2 h (Fig. 6). This suggests that the difference in oxalate accumulation between the still and shaken samples was minor. [^14^C]DHA exceeded [^14^C]AA in all extracts (Fig.6c–e): this of itself would not alter the nutritional value of the leaves since both DHA and AA serve as vitamin C in humans, but any DHA accumulated in the leaves may be prone to irreversible loss as oxalates and DKG. Equally, the difference between ascorbate loss in leaf discs (Fig.5c) was minimal between still and shaken samples.

Some OxT, previously reported to be a major product of DHA oxidation (Green and Fry, 2005, Parsons et al., 2011) was also formed in the spinach leaf discs. The content of OxT did not alter significantly over time or with the severity of the washing procedure (Fig.6b–d). The presence of OxT is notable as this is the first demonstration of OxT formation in planta, the compound previously being identified in vitro and in rose cell-suspension culture (Green & Fry, 2005). The presence of oxalate and OxT shows that the DHA has undergone oxidation. This in turn suggests that oxidative stress is causing the loss of ascorbate during washing.

The mechanical stress, caused by turbulent water, could lead to cellular wounding, which may lead to the production of various reactive oxygen species; ascorbate could then be consumed during the quenching of these reactive oxygen species, in its role as an antioxidant.

In order to avoid the loss of ascorbate during the commercial washing of pre-packaged salads, specifically spinach, it would be recommended that mechanical agitation should be minimised.

## 4 Discussion

The post-harvest storage of salad leaves has been fairly extensively studied in terms of nutritional factors such as ascorbate, but the loss of ascorbate during the washing process itself has been much less thoroughly characterised.

All the leaves tested in this study showed some degree of ascorbate loss during post-harvest storage. The 10-day storage period used here is slightly longer than the recommended storage time (the ‘best before end’ date is generally 5–7 days after packaging, rather than 10 days), but this time period is often used to study the effects of post-harvest storage time (Wagstaff et al., 2007).

Spinach leaves were found to be more susceptible to the loss of ascorbate during post-harvest processing than watercress or wild rocket leaves. Although cutting has been shown to negatively impact the ascorbate content of spinach (Cocetta et al., 2014, Lee and Kader, 2000), washing in various sanitisers has had inconclusive results (Cefola and Pace, 2015, Karaca and Velioglu, 2014). Many studies into post-harvest processing of spinach have involved the influence of storage temperature and storage time but the packing process had not been investigated in further detail for effects on ascorbate retention.

Our experiments showed that the mechanical stress experienced by spinach leaves during washing led to a significant loss of ascorbate, whereas mere submersion of the leaves did not significantly affect the ascorbate content. Further investigation demonstrated that ascorbate undergoes oxidation during spinach washing, producing oxalate. Spinach leaves are known to accumulate oxalate (Yang & Loewus, 1975), and oxalate can act as an anti-nutrient, as it insolubilises calcium, potentially leading to calcium deficiency and to the formation of kidney stones (Franceschi and Nakata, 2005, Libert and Franceschi, 1987, Noonan and Savage, 1999).

The oxidative stress within leaves is known to be increased by mechanical stress and by processes such as cutting prior to the washing procedure (Cocetta et al., 2014). The use of leaf discs rather than whole leaves in some of our experiments could have affected the oxidation state of the samples, potentially with some oxidation being due to a wound response, as well as to the washing. Interestingly, wounding was demonstrated to increase ascorbate levels in *Arabidopsis* but decrease levels in tomato leaves; this decrease in ascorbate was attributed to an increase in oxidation by H_2_O_2_ (Suza et al., 2010). Oxalate is known to be produced from ascorbate during the reaction with H_2_O_2_, although in vitro oxalate was a fairly minor product, generated in smaller amounts than OxT (Green and Fry, 2005, Parsons et al., 2011), whereas it was the major product as observed in spinach leaves (Fig. 6). This difference is likely to be due to the presence of oxalyl esterase activity in vivo, rapidly converting initially formed OxT to oxalate (Green & Fry, 2005).

Pre-harvest factors, such as the growth stage at harvest can also affect the ascorbate content of salad leaves (Lee & Kader, 2000). This was verified in the current study, in which spinach leaves harvested at earlier growth stages showed an increased retention of ascorbate during a 10-day post-harvest storage period. Younger leaves are known to have higher ascorbate concentrations, reflecting the role of ascorbate in plant growth (Bergquist et al., 2006). Although the initial concentrations of ascorbate did not differ between juvenile and mature spinach leaves, it is possible that the younger leaves were more efficient at recycling ascorbate, and thereby less prone to irreversibly losing it.

The washing process used by Vitacress Ltd is unusual in the pre-packaged salad business as it does not use any chlorine, ozone or other anti-microbial agent; instead the leaves are washed only in single pass spring water. Ozone and chlorine have previously been shown to negatively affect the ascorbate content of salad leaves, including rocket and iceberg lettuce (Karaca and Velioglu, 2014, Kenny and O’Beirne, 2009, Martínez-Sánchez et al., 2006). Conversely, it was reported that there was no difference in the ascorbate content of cabbage when washed in the presence of ozone or chlorine compared with pure water (da Silva et al., 2015). The literature regarding the effect of chlorine washing on spinach is not conclusive. In contrast to results reported by Karaca and Velioglu (2014), the current study shows a significant loss of ascorbate when spinach is washed in the presence of chlorine rather than in pure water.

It has recently been suggested that the levels of chlorate residue on post-harvest processed salad leaves should be minimised (Gil, Marín, Andujar, & Allende, 2016). This could be done by using more dilute chlorine solutions or using an alternative sanitiser. Alternative substances have been investigated for their efficacy as suitable washing treatments for pre-packaged salad. Citric acid was shown to preserve vitamin C content of lamb’s lettuce more effectively than exogenous ascorbate treatment (Cocetta, Francini, Trivelline, & Ferrante, 2016). Equally oxalic acid was shown to improve the retention of antioxidant activity in both rocket and spinach during postharvest storage when compared to sodium hypochlorite treatment, however a water-only treatment (as in the current study) was not included (Cefola & Pace, 2015). Spinach leaves washed in calcium chloride showed greater vitamin C loss than control leaves or leaves washed in calcium lactate (Oliveira, Amaro, de Sain, & Pintado, 2016).

Although OxT concentrations did not alter with the different washing treatments, the presence of OxT is still noteworthy, as this is the first demonstration of its presence in spinach, though it has recently been detected in tomato (Truffault et al., 2017). OxT had previously been found to be produced from radiolabelled ascorbate in rose cell-suspension culture (Green and Fry, 2005, Parsons et al., 2011).

## 5 Conclusion

Spinach leaves are more susceptible to loss of ascorbate during the washing process than wild rocket or watercress. The loss of ascorbate would seem to be primarily due to mechanical stress, which manifests as oxidative stress, causing the oxidation of ascorbate. This ultimately leads to an increase in oxalate, an anti-nutrient. These findings are of commercial importance as changes to washing procedures could result in greater retention of ascorbate during postharvest storage.

## Conflict of interests

The authors declare that there is no conflict of interest regarding the publication of this paper.
