# Supplementary material for: Novel insights into ascorbate retention and degradation during the washing and post-harvest storage of spinach and other salad leaves
Source: Food Chem. 2017 Oct 15;233:237–46. doi: 10.1016/j.foodchem.2017.04.082 (PMC5441274; doi:10.1016/j.foodchem.2017.04.082)
Supplement: Supplementary Figs. S1 and S2 [file mmc1.pptx]

## Slide 1
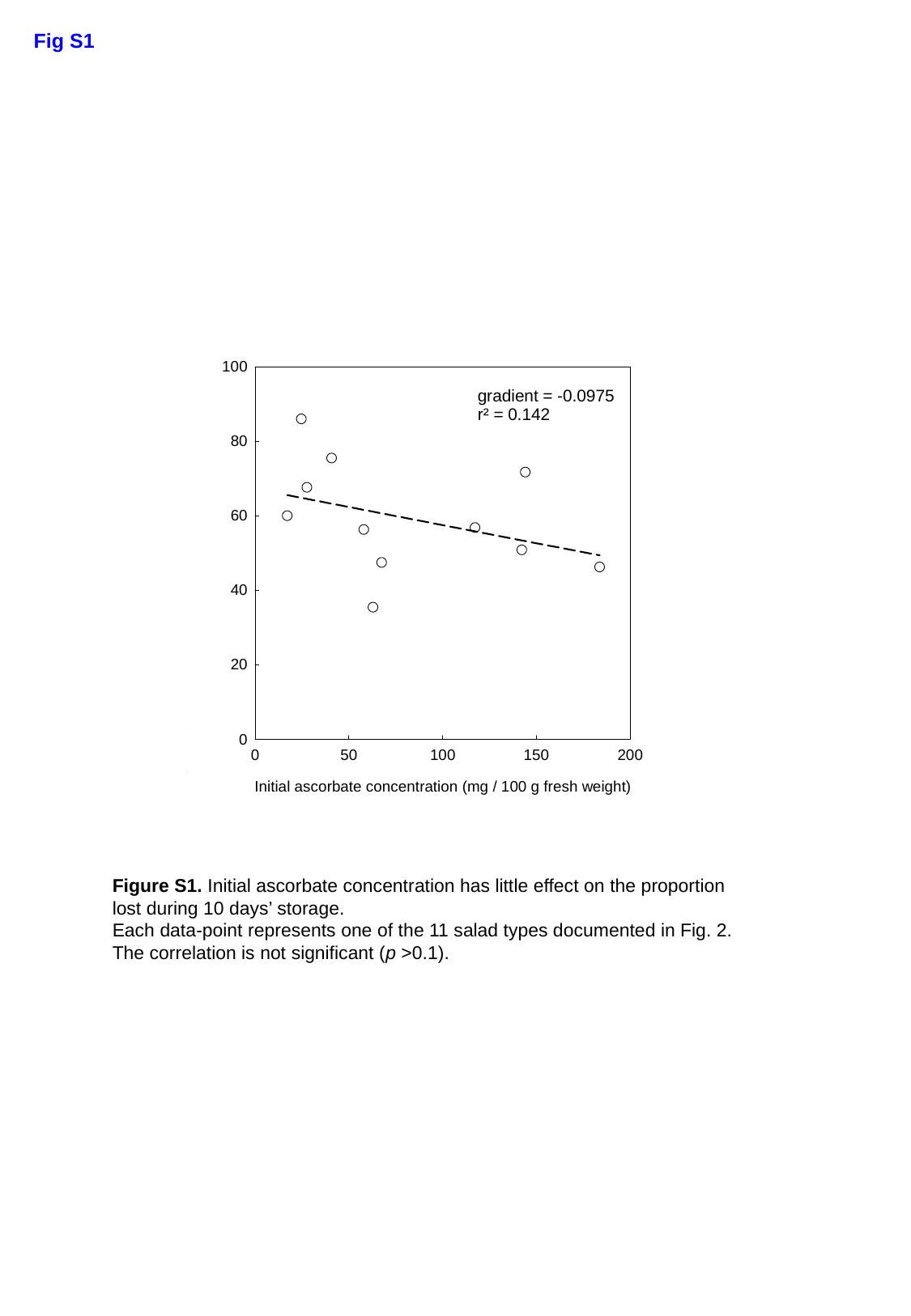

Fig S1
Figure S1. Initial ascorbate concentration has little effect on the proportion lost during 10 days’ storage.
Each data-point represents one of the 11 salad types documented in Fig. 2. The correlation is not significant (p >0.1).

## Slide 2
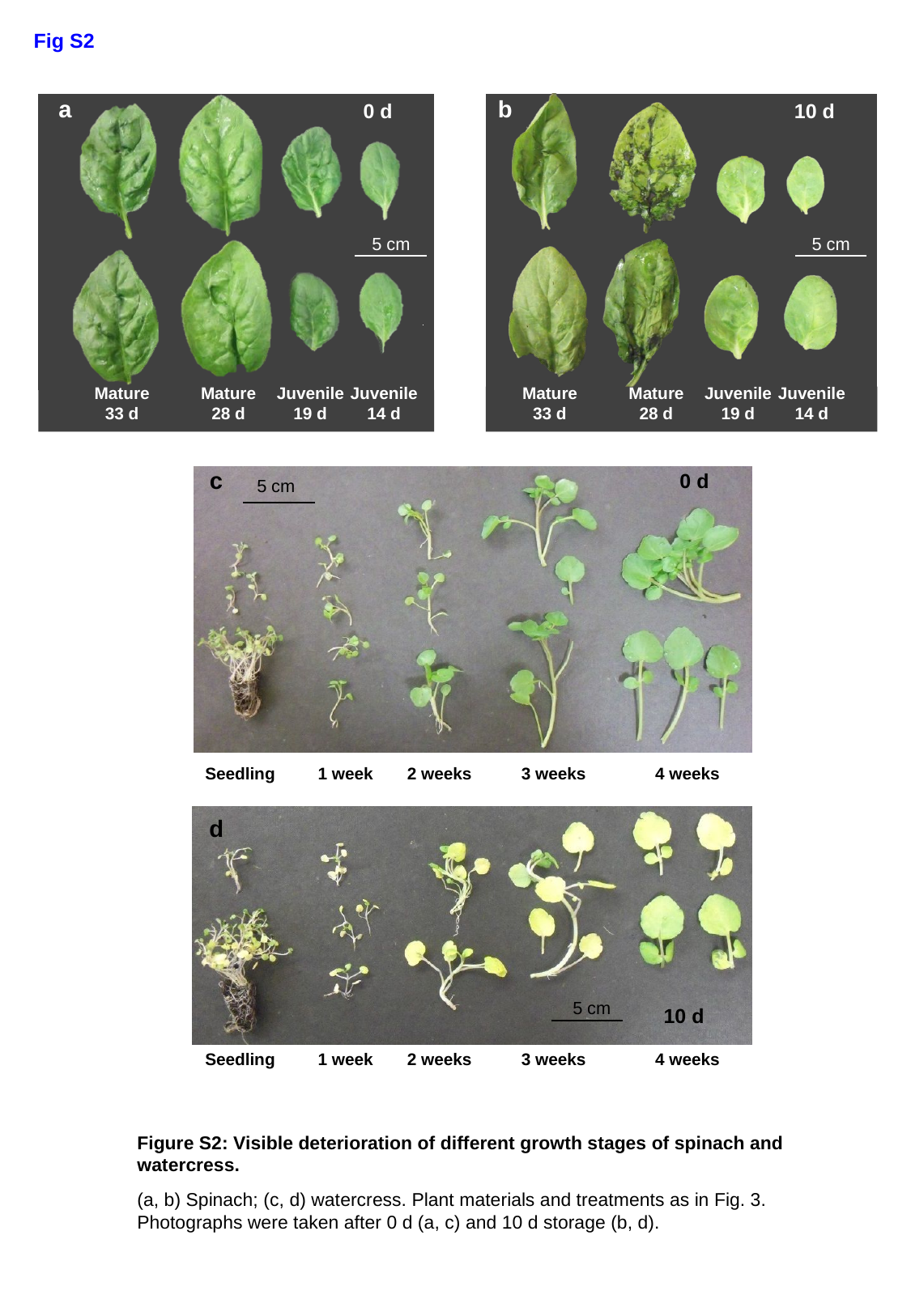

Fig S2
a
b
0 d
10 d
5 cm
5 cm
Mature 33 d
Mature 28 d
Juvenile 19 d
Juvenile 14 d
Mature 33 d
Mature 28 d
Juvenile 19 d
Juvenile 14 d
c
0 d
5 cm
Seedling
1 week
2 weeks
3 weeks
4 weeks
10 d
d
5 cm
Seedling
1 week
2 weeks
3 weeks
4 weeks
Figure S2: Visible deterioration of different growth stages of spinach and watercress.
(a, b) Spinach; (c, d) watercress. Plant materials and treatments as in Fig. 3. Photographs were taken after 0 d (a, c) and 10 d storage (b, d).
